# Promoting Inclusive Cancer Care: Beyond Medical Interpreters

**DOI:** 10.1093/oncolo/oyad290

**Published:** 2023-10-20

**Authors:** Alex G Thomas, Alyx B Porter

**Affiliations:** University of Chicago Pritzker School of Medicine, Chicago IL, USA; Division of Hematology/Oncology, Department of Neurology, Mayo Clinic, Phoenix, AZ, USA

**Keywords:** patient navigation, cultural sensitivity, ethnic and racial minorities, community networks

## Abstract

Opportunities exist to promote inclusive cancer care and clinical research to reduce disparity and achieve health equity. Through raising awareness of the nuances of the clinical encounter to promote inclusivity as well as reducing barriers to access and enrollment in clinical trials, a more complete understanding regarding the spectrum of disease and opportunity for inclusive cancer care can be achieved.

## Introduction

As defined by the National Institutes of Health (NIH), Black/African-Americans, Hispanic/Latino, American Indian/Alaska Native, Asian American, Native Hawaiians and other Pacific Islanders, socioeconomically disadvantaged populations, underserved rural populations, and sexual and gender minorities have received disparate healthcare and remain at continued risk of marginalization.^1^ Opportunities exist to promote inclusive cancer care and clinical research to reduce disparity and achieve health equity. Through raising awareness of the nuances of the clinical encounter to promote inclusivity as well as reducing barriers to access and enrollment in clinical trials, a more complete understanding regarding the spectrum of disease and opportunity for inclusive cancer care can be achieved. Enhancing resources such as patient and physician education, patient navigator programs, and community virtual support groups to be inclusive of diverse populations is imperative to reduce barriers that have led to disparate outcomes related to prevention, navigation of diagnosis, treatment adherence, and clinical trial enrollment.

## Culturally Inclusive Education

Language is the most obvious barrier addressed in healthcare to ensure health literacy and understanding in patients from different backgrounds. More holistically, consideration must be given to inclusive practices to all elements of cancer care to ensure a sense of trust and belonging for diverse populations. The 2003 Institute of Medicine report on “Unequal Treatment: Confronting racial and ethnic disparities in health care,” clearly identified the role of cultural competency training in healthcare to reach the goal of eradicating health disparities.^2^ Cultural competency has been defined as the attitudes, knowledge, and skills necessary for providing quality care to diverse populations, seemingly suggesting that requirements have been met to care for individuals from a variety of cultures. The impossibility of being adequately knowledgeable of every culture which is ever-changing is evident. A patient-centered approach therefore requires cultural humility, beyond competence, which is defined as, “relinquish[ing] the role of expert [of the patient’s culture] to the patient, becoming the student of the patient with a conviction of the patient’s potential to be a full partner in the therapeutic alliance.”^3^ To demonstrate cultural humility, in addition to increasing workforce diversity, one must develop communication skills that engender trust to honor the varied elements of diversity. Cultural humility can be expounded by having a healthcare workforce that reflects the diversity of the population cared for. This presents a viable solution in the complex matrix that perpetuates health disparities. Workforce diversity is not only important for our patients and learners; but it also impacts our colleagues and our understanding of the spectrum of disease experienced through research.

Cultural issues affect every aspect of the clinical encounter. To honor the differences through education enables the best care to be provided to each and every patient every day. Gaps in communication between clinicians, patients, and families may result from bias and stereotyping among healthcare providers, patients’ use of complementary or alternative healing traditions, health and healing beliefs of patients and families, health literacy, historical distrust, and language barriers.^2^ Establishing a therapeutic alliance between the patient and physician founded upon trust enables potential improvement in time to diagnosis and leads to increased adherence to the treatment plan and optimally enhanced outcomes. While the use of a trained healthcare interpreter is essential to reducing language barriers, there are additional communication tools that clinicians must employ to establish trust and elicit a patient or family’s health and healing beliefs, such as LEARN (Listen. Explain. Acknowledge. Recommend. Negotiate.)^4^ and Kleinman’s questions.^5^ Optimizing communication within the encounter through listening, education, and then negotiation surrounding treatment recommendations is critical in ensuring compliance. Part of the negotiation includes acknowledgement of the patient’s health and healing beliefs within their own cultural context and a willingness to partner to holistically honor the patient.

In addition to educating healthcare workers on cultural competency, patient education is also an important component which requires greater efforts toward personalization and understanding. Promoting educational resources for patients with cancer that resonate with their diverse identities can improve understanding, adherence with screenings and treatment, and overall outcomes. For example, patients identifying as sexual and/or gender minorities (SGM) have benefitted from education providing reassurance on disclosure of these identities to providers and pertinent information (ie, cancer types that disproportionately affect SGM populations).^6^ In addition, storytelling presents a meaningful approach to humanizing information in both the African-American^7^ and Alaska Native^8^ communities. Pérez et al captured the stories of African-American breast cancer survivors (AABCS) who shared their experiences on coping and communicating side effects from their cancer treatment.^7^ African-American patients, reported feelings of representation, appreciation, and trustworthiness upon listening to stories of AABCS, in turn encouraging patients to complete follow-up mammography and providing emotional support.^7^ Another example of storytelling reflecting cultural sensitivity involves the Alaska Native community, involving a program focused on creating culturally respectful cancer education to improve the ability of Alaska community health workers (CHWs) to facilitate such education.^8^ Given storytelling’s importance in sharing knowledge in Alaska Native culture as well, this program encouraged community healthcare workers to create narrative media communicating preventative measures to reduce cancer risk and recommended cancer care resources.^8^ Storytelling made the topic of cancer easier to discuss, promoting greater ease in sharing personal experiences and encouraging open, culturally respectful communication as opposed to silent avoidance.^8^

## Patient Navigator Program Enhancement

Patient navigator programs, while an additional resource within the care team, have shown demonstrable improvements in mitigating barriers to improving compliance, access to cancer treatment and outcomes.^9,10^ In a traditional setting, the navigator may have a nursing background and is employed by the institution to support the patient through various elements of their care ranging from treatment to financial and social services. To enhance the quality of these programs, elements of diversity could be improved by flipping this traditional structure and incorporating community members as patient navigators/advocates to promote more inclusion and collaboration between healthcare institutions and the community served. Community engagement can be strengthened through building bridges through appropriate training of community members in core competencies in patient navigation to enhance awareness of cultural sensitivity and community norms/preferences into patient-centered care. Such a program would incorporate cultural elements native to the community, such as spiritual/religious support, providing educational opportunities to patients with cancer with community support systems in place that would improve patient care.

Incorporation of community members, who share similar racial/ethnic backgrounds with the patients served, into healthcare teams supports development of culturally, linguistically, and individually tailored interventions to support patients. The community members would be chosen by patients and trained to address burdens related to care. It is essential to have community members assist in fostering trust and collaboration, as some minority patients seeking cancer treatment are often disconnected from the hospital or supporting health systems^9,10^ due to historical distrust stemming from previous negative experiences in healthcare settings as well as other systemic, ingrained disparities. Community engagement celebrates and fosters not only the relationships surrounding the patients but also strengthens the relationship between the community and the healthcare institution.

## Community (Virtual) Support Groups

The forum of support groups provides emotional support and education on up-to-date cancer research. Support groups are widely used and accepted as best practice for patients with cancer to engage in sharing psychosocial support and sharing of resources. The psychosocial distress patients with cancer experience once diagnosed, while seeking treatment, and over the survivorship journey is significant.^11,12^ Promoting support groups for diagnosis-specifics is not enough to fully represent the whole patient experience when faced with a cancer diagnosis. To create a safe environment to foster connection and collaboration, patients should have access to diverse and inclusive support groups. While diagnosis relatability is necessary, patients from diverse backgrounds may experience a sense of isolation given limited representation in their local healthcare environments. Virtual support groups based on diagnosis and diversity identifiers are important to help patients of diverse backgrounds connect to providers and community resources. Strengthening the support group model through incorporating diversity and inclusion measures such as constructing identity-specific group topics for LGBTQIA+ or Veteran patients with cancer is imperative. Promoting support groups for diagnosis-specifics is not enough to fully represent the whole patient experience when faced with a cancer diagnosis. To create a safe environment to foster connection and collaboration, patients should have access to diverse and inclusive support groups. Affinity groups can also be an opportunity to study research outcomes like treatment adherence, distress monitoring, and other factors related to cancer research. Overall, support groups, virtual and in-person, have proven to be successful with addressing burdens related to care.

## Conclusion

Inclusive cancer care must go beyond traditional frameworks of care such as interpreter services, access, and treatment regimens as well as focus on how one’s personal identity and diverse communities that patients belong to are of central importance to providing care. Building upon physician and patient education, existing patient navigator programs, and community virtual support groups by taking cultural sensitivity and inclusion into greater consideration when creating these resources would be very impactful. These efforts underscore the importance of a more holistic approach to cancer care which strives toward creating more equitable and culturally respectful support systems for patients.
